# Diplopia as a sign of third nerve palsy due to intracranial aneurysm: a case report

**DOI:** 10.11604/pamj.2024.48.104.44251

**Published:** 2024-07-12

**Authors:** Restiana Hilda Islami, Lukisiari Agustini, Gatot Suhartono

**Affiliations:** 1 Department of Ophthalmology, Dr. Soetomo General Academic Hospital, Surabaya, Indonesia,; 2 Department of Ophthalmology, Faculty of Medicine, Universitas Airlangga, Surabaya, Indonesia

**Keywords:** Third nerve palsy, diplopia, ptosis, aneurysms, case report

## Abstract

Third nerve palsies that result from head injuries or intracranial aneurysms may sometimes show symptoms of aberrant regeneration and only partially heal. The usual characteristics of oculomotor nerve palsy, caused by compression of the third cranial nerve, are severe ptosis, deficiencies in elevation, depression, and adduction, and a dilated, poorly responding pupil on the afflicted side. The parasympathetic fibres are usually spared from a vasculopathic lesion and impacted by compressive lesions because they are situated in the peripheral segment of the oculomotor nerve as it leaves the brain stem. When pupillary mydriasis coexists with acute third cranial nerve palsy, an aneurysm at the junction of the internal carotid and posterior communicating arteries has to be thoroughly and quickly explored using the necessary neuroimaging techniques.

## Introduction

Third cranial nerve palsy can arise from an acquired or congenital condition. The majority of adult-acquired causes are ischemia-related and go away entirely in a few months. Both congenital and post-traumatic oculomotor nerve palsies resulting from cerebral aneurysms and head trauma can heal to some extent and occasionally show symptoms of abnormal regeneration [1]. The classic signs of oculomotor nerve palsy severe ptosis, abnormalities in elevation, depression, and adduction, and a dilated, poorly responding pupil on the afflicted side are caused by compression of the third cranial (oculomotor) nerve. The parasympathetic fibres are usually spared from a vasculopathic lesion (such as diabetes mellitus) and impacted by compressive lesions (such as tumours and aneurysms) because they are situated in the peripheral (superficial) segment of the oculomotor nerve as it leaves the brain stem. An aneurysm at the confluence of the internal carotid and posterior communicating arteries has to be thoroughly and quickly evaluated with suitable neuroimaging when acute third cranial nerve palsy is accompanied with pupillary mydriasis [2]. It is well recognised that cerebral aneurysms, particularly those of the IC-PC (internal carotid posterior communicating artery junction), cause oculomotor nerve palsy.

There are several possible causes of pupillary involvement in isolated third nerve palsy. The most frequent, potentially fatal cause is intracranial aneurysm [3,4]. According to reports, the incidence of palsy in IC-PC aneurysm ranges from 34% to 6%. Aneurysms are the most prevalent cause of isolated third nerve palsy. Rarely, temporary third nerve palsy can also be brought on by rapidly growing aneurysms. Increased intracranial pressure (ICP) due to clots, edema, or hydrocephalus, as well as hemorrhagic dissection of the nerve, were categorised as indirect causes of ONP, while midbrain hemorrhage, direct local pressure caused by aneurysms, and hemorrhagic dissection of the nerve were classified as direct causes. Increased intracranial pressure (ICP) due to clots, edoema, or hydrocephalus, which causes uncal herniation, was classified as an indirect cause of oculomotor nerve palsy, whereas midbrain haemorrhage, hemorrhagic dissection of the nerve, and direct local pressure by aneurysms were considered as direct causes. Venous ischemia with neurological signs and symptoms can also result from high pressure in the venous drainage system [5]. In this case report, we will describe a 58-year-old female patient who came with diplopia and unilateral ptosis also dilated pupil as the manifestation of oculomotor nerve palsy in which caused by an aneurysm. The writing of this case aims to have better understanding to address proper management and to remind that ocular manifestation can be a sign of intracranial aneurysm.

## Patient and observation

**Patient information:** a 58-year-old woman came to the outpatient and complained of diplopia and ptosis. The patient came with a chief complaint of double vision 2 months ago followed by ptosis in the right eye, headache in the right side, and no history of eye redness, vomiting or nausea. She admitted that she had hypertension but never took medicine. Neither immunodeficiency disorders nor diabetes have a history in the family. The patient denied a history of traumatic events or accidents. The patient was previously treated with oral analgetic from a general practitioner without significant improvement. Then referred to the neurosurgery department.

**Figure 1:**
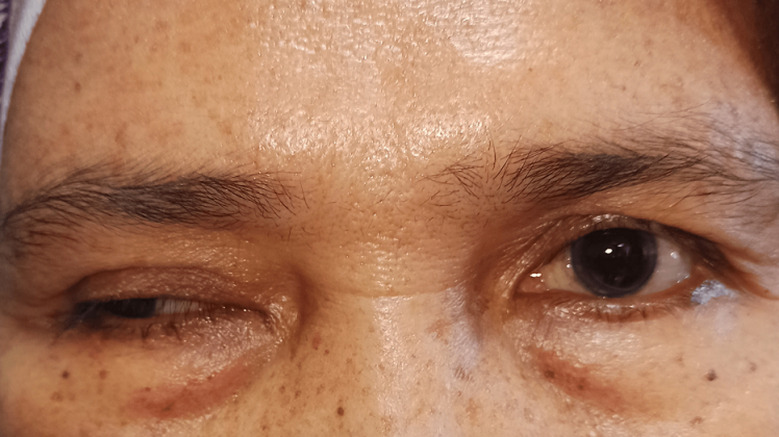
a 58-year-old woman with double vision, ptosis and eye deviation to inferolateral

**Figure 2:**
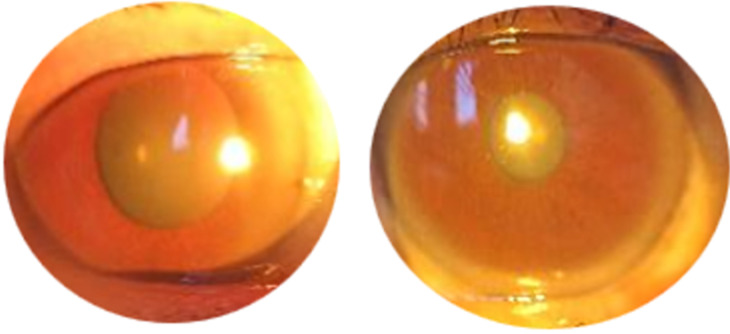
anisocoria of right and left pupil

**Clinical findings:** from the examination of vital signs, an increase in blood pressure was detected. During her first visit, examination revealed visual acuity of the right eye was 0.3 pinhole with no improvement, and the left eye was 0.33 with pinhole became 0.5. Color vision examination and intraocular pressure were within normal limits. The anterior segment showed ptosis in the right eye (Figure 1) and from anterior segment examination, we detected rounded pupils and anisocoria, with a diameter of 6 mm in the right eye and 3 mm in the left eye (Figure 2). Light reflex was normal in the left eye but there were no reflexes found in the right eye with direct light stimulus. Ocular motility in the right eye found limitation -4 to supero and infero lateral and -4 to superior, superomedial, medial, inferomedial, inferior, but there was no limitation on her left eye when she gazed in a lateral direction. From the left eye, there was limitation -1 in medial and infero medial movement, but there was no limitation on her left in the other direction. There was no pain of ocular movement in all gaze.

**Figure 3:**
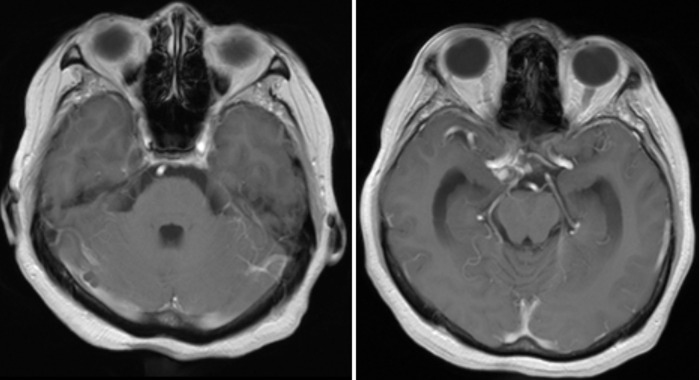
aneurysm on the right Middle cerebral artery (MCA) with an aneurysm pocket showing the base of the aneurysm merging with the right posterior communicating artery, pressing the right oculomotor nerve

**Figure 4:**
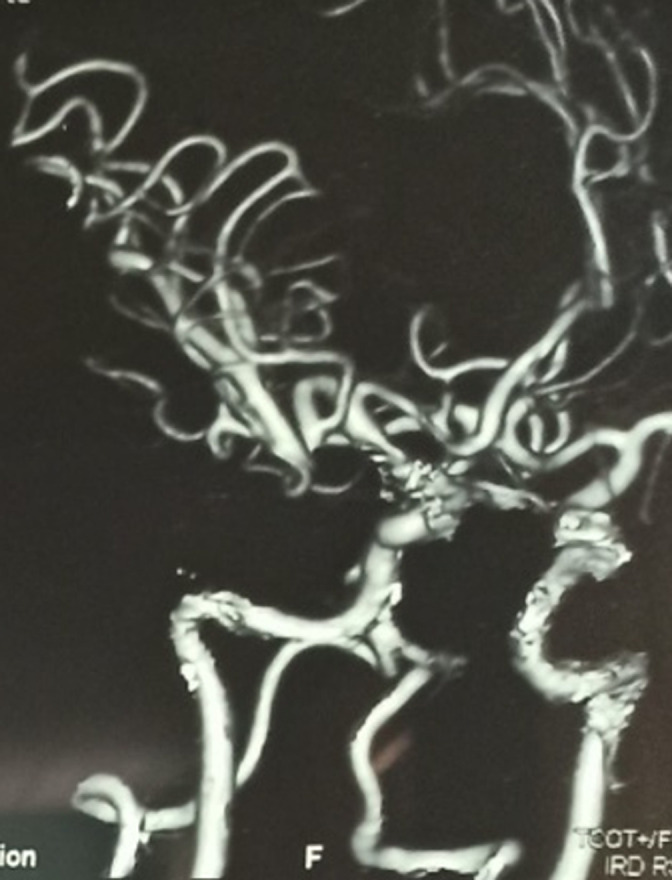
magnetic resonance angiography circullus Willis: visible saccular aneurysm in the right Posterior communicating artery

**Figure 5:**
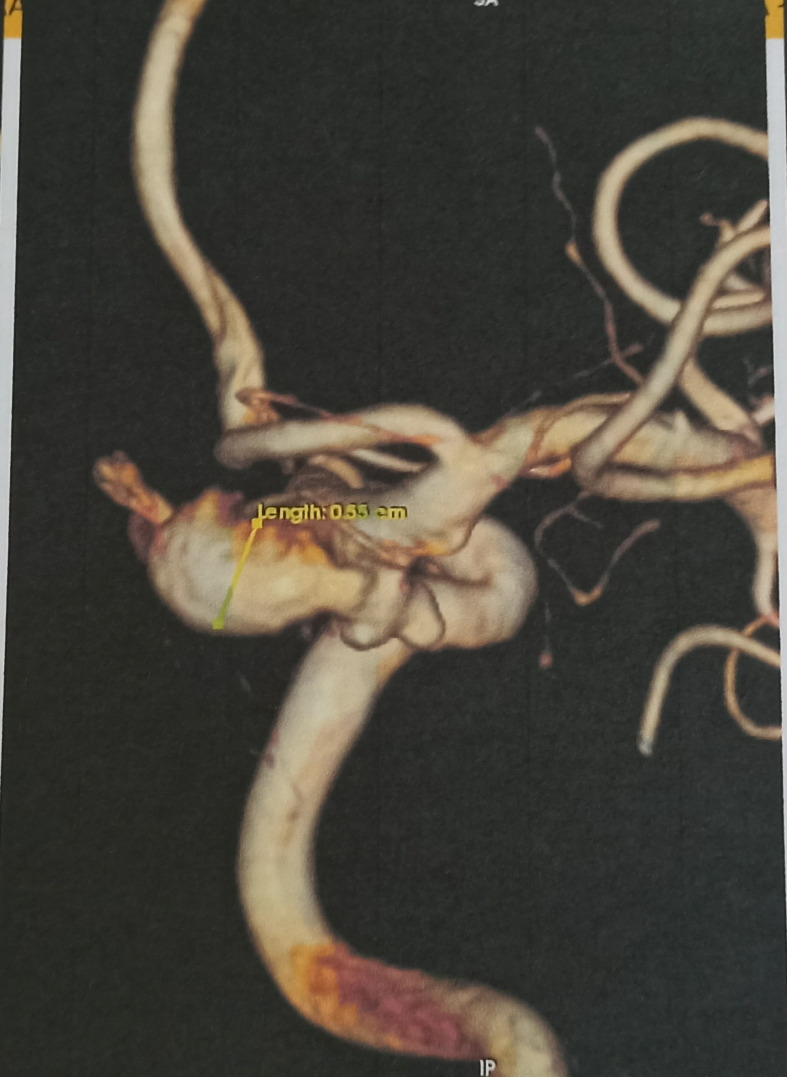
3D CT angiography found the size of aneurysm in the neck

**Diagnostic assessment:** brain Magnetic Resonance Imaging (MRI) with contrast was performed due to suspicion of intracranial lesion. MRI showed a saccular aneurysm on the right middle cerebral artery (MCA) with an aneurysm pocket (neck) measuring ± 0.16 cm head size: height ± 0.38 cm width ± 0.36 cm shows the base of the aneurysm merging with the right posterior communicating artery, pressing right oculomotor nerve (Figure 3). From the magnetic resonance angiography (MRA) circullus Willis looks patent, visible saccular aneurysm in the right posterior communicating artery (PCOM) (Figure 4). 3D CT Angiography showed the size of the aneurysm the neck was 2.7 mm, the height was 11 mm, and the dome 5.5 mm (Figure 5).

**Diagnosis:** oculomotor nerve palsy caused by right PCOM aneurysm.

**Therapeutic interventions:** in this patient, an aneurysm clipping was performed and the patient was followed up the next month at the outpatient clinic.

**Figure 6:**
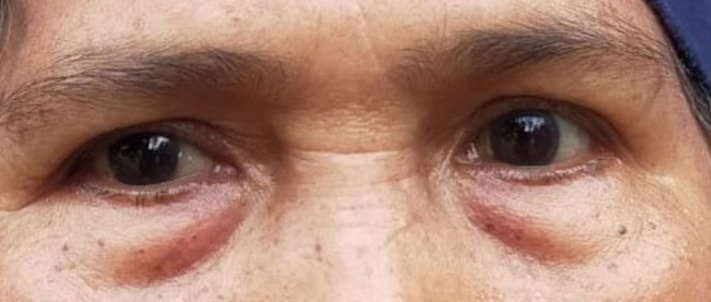
follow-up five months after treatment revealing normal primary gaze with no ptosis

**Follow-up and outcome of interventions:** one month later, she was controlled at the outpatient clinic after undergoing a clipping aneurysm surgery. She said that her eyelids can open a little more than before, there is no double vision, her right and left eye visual acuity improved to 0.5, her pinhole to 0.8, and her left eye became 0.8. Because of the COVID-19 pandemic, the patient was followed up via telemedicine where she showed pictures of her eye gaze. Five months following the aneurysm clipping, we observed significant changes. We observed that the eye's primary position was central, and the restriction on eyeball movement had decreased (Figure 6).

**Patient perspective:** “I never thought I would get sick like this, the enlargement of the blood vessels in my brain really scared me. Luckily my illness was treated immediately and action was taken so that my eyes returned to normal.”

**Informed consent:** the patient and family were acknowledged about the case reported and agreed that the case would be published for the benefit of people and medicine.

## Discussion

Oculomotor nerve palsy accompanied by pupil dilation and ptosis often arises from an aneurysmal lesion. Third cranial nerve (CN III) palsies can result in impairment of both the somatic muscles (including the superior, inferior, and medial recti; inferior oblique; and levator palpebrae superioris) and the autonomic muscles (namely the pupillary sphincter and ciliary muscles). A full CN III palsy is characterized by the eye being deviated downwards and outwards, total drooping of the eyelid, and the inability to move the eye inwards, downwards, or upwards. The pupil may or may not be experiencing a full third cranial nerve palsy [6,7].

Intracranial aneurysms are the primary cause of solitary oculomotor nerve palsy with pupillary involvement, especially in patients who have had acute and severe pain in or around the eye. Aneurysms often originate from the point where the internal carotid artery and posterior communicating artery meet. However, aneurysms found at the apex of the basilar artery or the intersection of the basilar artery and superior cerebellar artery can also cause comparable clinical symptoms. Aneurysms can cause damage to the oculomotor nerve by direct compression, a minor bleed, or during a significant rupture [8].

Patients may have varying levels of dysfunction in the levator palpebrae or extraocular muscles. Aneurysms occurring at the junction of the posterior communicating artery (PCoA) and internal carotid artery (ICA) are located next to cranial nerve III (CN III) and can cause CN III palsy as the first sign of aneurysm growth or rupture. Pupillary involvement arises due to the location of the pupillomotor fibers, which are situated near the PCoA on the medial side of the nerve. Therefore, in the absence of contrary data, the physician should presume that a nontraumatic CN III palsy with pupillary involvement or signs of progressing to pupillary involvement is caused by an aneurysm. Emergent cerebrovascular imaging, such as catheter angiography, magnetic resonance angiography (MRA), or computed tomography angiography (CTA), should be performed based on the clinical circumstances and consultation with a neuroradiologist. The angiographic techniques employed may effectively identify nearly all aneurysms located in this area that result in a CN III palsy. State-of-the-art CTA and MRA procedures have a high level of accuracy in detecting aneurysms with a diameter as small as 3 mm. Between these two technologies, CTA is more rapid offers pictures with somewhat higher quality, and might potentially reveal signs of a subarachnoid hemorrhage. Magnetic resonance imaging (MRI) performed as part of routine examination using the MRA technique has a higher probability of detecting non-aneurysmal lesions [6]. Our patients have ptosis, and anisocoria as well as limitation of the movement of the eyeball caused by an aneurysm of the PCOM.

The current gold standard for diagnosing cerebral aneurysms is intra-arterial digital subtraction angiography (IADSA), however, computed tomography angiography (CTA) and magnetic resonance angiography (MRA) can also be used for diagnosis. During IADSA, the application of contrast allows the aneurysm to be seen on fluoroscopy as a radio-opaque, smooth-margined, saccular out-pouching of the cerebral vasculature. The presence of a blood clot within the aneurysm will appear as a disruption in the smooth edge due to the inability of contrast material to pass through the stagnant, clotted blood. CTA or MRA is more effective than IADSA in clearly showing a thrombus, as these imaging techniques provide detailed visualization of the vessel wall and the surrounding area, not only the inner space of the vessel. During a computed tomography angiography (CTA), if the aneurysm is sufficiently enough, it will be visible as a rounded, spherical mass with the same level of whiteness as the main blood arteries [9]. In this case, these patients are only undergoing CT angiography and MR Angiography because IADSA is not available as the gold standard for vascular malformations in our hospital.

The majority of acquired causes in adults are a result of ischemia and often recover entirely within a few months. When dealing with pupil-sparing palsy, it is important to determine whether the patient has diabetes or hypertension, since these conditions might have an impact on both the prognosis and the anesthetic considerations. It is crucial to closely observe the development of pupil engagement during the initial days following the start of strabismus. The patient is advised to promptly seek medical attention if they experience worsening photophobia in the afflicted eye or any new visual impairment when reading. Patients and their family members are advised to watch out for anisocoria. Patients are advised to promptly seek medical attention if they have any further neurological problems. Before undertaking surgical procedures for strabismus, it is imperative to first address the underlying systemic reasons that are currently active. It is crucial to maintain strict control of diabetes and hypertension. It is important to effectively manage hyperlipidemia, anemia, and obesity. Neurosurgical intervention may be necessary to treat a brain tumor or an aneurysm. Typically, surgery for strabismus is postponed for at least 6 months after the start of oculomotor palsy. If there is further progress, the procedure might be postponed even longer [10]. Our patients have had a clipping surgery and 5 months after surgery the movement of the eyeball has improved so there is no need for a strabismus surgery.

Surgical intervention is typically necessary for oculomotor palsy caused by trauma or cerebral lesions. However, there is a possibility of spontaneous improvement, particularly following the removal of a tumor or aneurysm. Following surgical aneurysm repair, the recovery of third cranial nerve function often proceeds as follows: increased function of the levator palpebrae and medial rectus precedes, and is occasionally followed by improved pupillary functions. This case is an elderly woman who developed oculomotor nerve palsy, while not having any previously diagnosed systemic illness. Oculomotor nerve palsy necessitates imaging evaluation, which reveals the existence of an aneurysm. This case demonstrates the amelioration of an ocular aneurysm with prompt treatment, resulting in favorable outcomes.

## Conclusion

Oculomotor nerve palsy is one of the signs of intracranial abnormalities and may warrant further imaging. By identifying the condition at an early stage and taking prompt action, we can effectively avoid the progression and morbidity. Vision and eye function can be restored to their normal state.
